# The Expression of Kisspeptin Receptor, Catsper 3 and Acrosome Integrity in Oligozoospermic and Normozoospermic Individuals

**DOI:** 10.3390/genes17030266

**Published:** 2026-02-26

**Authors:** Nejat Ünlükal, Seda Aniç, Duygu Dursunoğlu, Seda Şimşek, Muslu Kazım Körez, Abdullah Şentürk

**Affiliations:** 1 Department of Histology and Embryology, Medicine Faculty, Selcuk University, Konya 42700, Turkey; sedaberen17@gmail.com (S.A.); sedaatay89@gmail.com (S.Ş.); abdullahsenturkbioeng@gmail.com (A.Ş.); 2 Department of Biostatistics, Medicine Faculty, Selcuk University, Konya 42700, Turkey; mkkorez@gmail.com

**Keywords:** oligozoospermia, CatSper3, Acrosome, male infertility, kisspeptin, KISS1R

## Abstract

**Background**: Male infertility remains a significant clinical challenge. The KISS1R-CATSPER3 signaling axis and acrosomal integrity are vital for fertilization, yet their regional expression patterns in subfertile men are not fully characterized. **Objectives**: This study investigated regional expression patterns of KISS1R and CATSPER3 and evaluated acrosomal integrity in oligozoospermic and normozoospermic individuals, accounting for demographic confounders. **Methods**: A total of 52 participants were selected from 568 candidates and divided into normozoospermic (*n* = 26) and oligozoospermic (*n* = 26) groups. Analysis included qPCR and immunofluorescence for KISS1R and CATSPER3. Regional expression was independently scored by blinded researchers. Statistical models were adjusted for age and body mass index (BMI). **Results**: Acrosomal integrity was significantly lower in the oligozoospermic group (*p* < 0.001). After adjusting for age and BMI, overall protein expression of CATSPER3 and KISS1R remained significantly lower in oligozoospermic men (*p* < 0.05). Regional analysis showed that CATSPER3 head staining differences disappeared after adjustment, whereas lower tail expression persisted. Higher KISS1R head expression in oligozoospermic men remained significant. qPCR showed no differences in KISS1R or CATSPER3 mRNA levels (*p* > 0.05), indicating post-translational regulation. **Conclusions**: KISS1R-CATSPER3 axis downregulation is a hallmark of oligozoospermia that persists independently of age and BMI. Regional protein instability, despite stable mRNA levels, suggests that post-translational regulation is critical for fertility. These markers may serve as potential diagnostic or therapeutic indicators for male infertility.

## 1. Introduction

Infertility is a significant reproductive health problem affecting approximately 15% of couples worldwide [1]. Male infertility contributes to about 50% of infertility cases and is solely responsible for 25–30% of them [2]. The diagnosis of male infertility is primarily based on the spermiogram test, which evaluates sperm count, motility, vitality, and morphology according to the criteria defined by the World Health Organization (WHO) [3]. However, successful in vitro fertilization (IVF) outcomes have been associated with sperm DNA integrity, independent of conventional semen parameters, highlighting the need for additional markers beyond standard spermiogram analyses [4].

After sperm cells are produced in the testes, they undergo epididymal maturation, acquiring characteristics such as progressive motility, chromatin protamination, and tight packaging to ensure DNA stability, which are critical for fertilization potential [5]. Further fertilization abilities are gained through capacitation in the female genital tract, a process that involves hyperactivated motility and the acrosome reaction [6]. Calcium ions (Ca^2+^) play a central role in both epididymal maturation and capacitation, and their uptake into spermatozoa occurs through several pathways, including the cation channel of sperm (CATSPER, specifically CATSPER3) and the kisspeptin receptor (KISS1R) signaling axis [7,8].

CATSPER calcium ion channels are the primary mechanism for calcium transport from the extracellular space into sperm, particularly the flagellum. They are essential for sperm hyperactivation, which enables spermatozoa to penetrate the oocyte membrane [9]. Defects or loss of any CATSPER subunit can result in impaired sperm function and infertility [9,10]. CATSPER3, a member of this family, has been implicated in sperm motility and fertilization capacity.

Kisspeptin (KISS1) and its receptor (KISS1R) represent another pathway for calcium regulation in sperm. Both molecules are expressed in the equatorial segment, which is critical for sperm–oocyte fusion [11,12], as well as in the mid-tail region, which is important for calcium storage [13,14,15]. They have also been detected in the head, neck, and tail regions of human spermatozoa [16]. KISS1 increases sperm motility by elevating intracellular calcium and promoting hyperactivation. Seminal kisspeptin levels correlate positively with sperm concentration, total sperm count, and motile sperm count [17], while serum kisspeptin levels are significantly lower in infertile men compared to fertile men [18].

Acrosomal integrity is another key determinant of intracellular calcium dynamics in spermatozoa. The acrosome serves as a calcium source necessary for the acrosome reaction, with extracellular calcium and the IP3 (inositol triphosphate) pathway facilitating exocytosis. Changes in ion permeability are required to achieve the hyperpolarization necessary for acrosome activation, and non-capacitated sperm with depolarized membranes are unable to undergo this reaction [19]. This highlights the importance of acrosomal integrity for proper sperm function.

The aim of the present study was to comprehensively investigate the regional expression patterns of KISS1R and CATSPER3 in human spermatozoa and to evaluate their potential relationship with acrosomal integrity in oligozoospermic individuals compared to normozoospermic controls. We hypothesized that the downregulation of the KISS1R-CATSPER3 signaling axis, particularly in the sperm head region, is associated with impaired acrosomal stability. This molecular deficit may serve as a potential indicator of reduced fertilization capacity, contributing to the underlying pathophysiology of oligozoospermia beyond simple reductions in cell count.

## 2. Materials and Methods

### 2.1. Collection of Semen Samples, Semen Analysis and Determination of Groups

**Figure 1:**
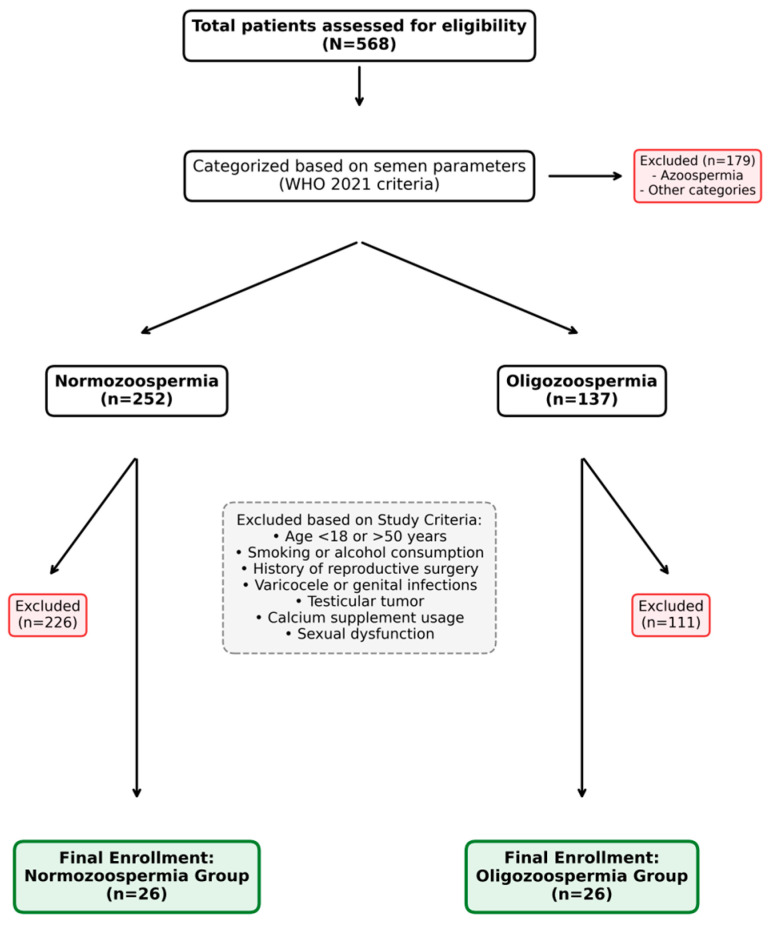
Flow chart of the participant recruitment and selection process according to WHO 2021 criteria [3] and specific study exclusion factors.

In this study, 52 volunteers between the ages of 18 and 50 years were enrolled who applied to the IVF Centre Andrology Laboratory of Selçuk University Medical Faculty for standard semen analysis. The study included subjects who reported regular sexual activity and no history of sexual dysfunction; who did not have a testicular tumor, varicocele, a genital tract infection or azoospermia; who had not undergone surgery affecting/inhibiting reproductive function; who did not smoke or drink alcohol; and who did not take calcium supplements. Informed consent was obtained from all participants. The recruitment, exclusion, and final participant selection process is summarized in the study flow chart (Figure 1).

Semen parameters evaluated according to the World Health Organization (WHO) 2021 guidelines included sperm concentration, total sperm count, total sperm motility, progressive sperm motility, sperm vitality and normal sperm morphology. The subjects were divided into two distinct study groups based on the reference values for semen parameters specified in the WHO 2021 guidelines: normozoospermia and oligozoospermia. Normozoospermia group: Sperm concentration ≥ 16 million/mL, total sperm count ≥ 39 million, total sperm motility ≥ 42%, progressive sperm motility ≥ 30%, sperm vitality ≥ 54% and normal morphology of sperm count ≥ 4% (Kruger Strict criterion). Oligozoospermia group: Sperm concentration < 16 million/mL and total sperm count < 39 million, total sperm motility ≥ 42%, progressive sperm motility ≥ 30%, sperm vitality ≥ 54% and normal morphology of sperm count ≥ 4% [3].

Semen samples were collected by masturbation after 2–7 days of sexual abstinence. Semen samples were liquefied at 37 °C for 30–60 min. Sperm concentration and motility characteristics of samples with a measured volume were evaluated using a Makler counting chamber with a phase-contrast microscope at 200–400× magnification. Sperm motility was classified as total sperm motility (WHO class A + B + C) and progressive motility (WHO class A + B). Total sperm count was calculated by multiplying sperm concentration by semen volume.

For morphological examination, smear preparations were made from the semen samples and allowed to air dry. The preparations were then fixed in methanol and stained with a Diff-Quick stain as described previously. At least 200 stained spermatozoa per semen sample were examined by oil immersion under bright field optics at ×1000 magnification. The percentage of normal sperm morphology was calculated as the number of spermatozoa with normal morphology divided by the total number of spermatozoa. Sperm viability was assessed by eosin–nigrosin staining. The stained semen samples were evaluated under an Olympus CX21 microscope (Olympus, Tokyo, Japan) on a minimum of 200 spermatozoa per semen sample. The percentage of sperm viability was calculated as the ratio of viable cells to total cells.

This study was conducted in accordance with the Declaration of Helsinki, and the study design was approved by the Research Ethical Committee of the Faculty of Medicine Ethics Committee, Selcuk University, Turkey (approval number: 2022/283).

### 2.2. Histological Analyses

Sperm samples were spread onto poly-L-lysine-coated slides and dried. The preparations prepared for the evaluation of CATSPER3 expression in normozoospermic and oligozoospermic individuals were labeled by the immunofluorescence method using anti-CATSPER3 (rabbit polyclonal antibodies—bs-7136R unconjugated, Thermo Fisher Scientific, Waltham, MA, USA) and anti-KISS1R primary antibodies (rabbit polyclonal antibodies—STJ93845 unconjugated, Thermo Fisher Scientific, Waltham, MA, USA) at a 1/100 dilution. Donkey anti-rabbit IgG-FITC (sc-2090, Santa Cruz Biotechnology, Dallas, TX, USA) was used as the secondary antibody at a 1/200 dilution. The sections were then coated with mounting medium containing DAPI. All immunohistochemical procedures were carried out in a dark and humid environment. An Olympus BX51 trinocular fluorescence microscope (Tokyo, Japan) was used for examination. To evaluate regional expression patterns, spermatozoa were compartmentalized into three distinct anatomical regions: the head, neck, and tail. The immunofluorescence signals were independently evaluated by two experienced observers who were blinded to the study groups. Only spermatozoa displaying moderate to high intensity fluorescence, clearly distinguishable from the background noise and negative controls, were considered positive for expression. Ten different areas were randomly selected from sperm smear preparations at 40× and 100× magnification. Expression percentages were calculated by dividing the number of labelled cells (total and region-specific) in photographs taken with a Olympus DP72 digital camera (Tokyo, Japan) by the total number of sperm counted (at least 200 cells per slide).

### 2.3. Acrosomal Integrity

FITC (fluorescein isothiocyanate) conjugated peanut glutinin (PNA), FITC-PNA (L7381 Sigma, Burlington, MA, USA), which is one of the most commonly used lectins in immunofluorescence labeling, was used to assess acrosome membrane integrity.

Sperm samples were spread on a microscope slide, air dried and fixed with pure methanol cooled to −20 °C. They were incubated with PBS solution (100 µg/mL) containing FITC-PNA at 37 C0 for 30 min. At least 200 spermatozoa were photographed with a DP72 digital camera in 10 different randomly selected areas under a 100× objective.

Green fluorescence in the acrosomal region indicates an undamaged acrosomal membrane, whereas no staining in spermatozoa or green fluorescence only in the equatorial segment indicates a damaged acrosomal membrane. This is because the damaged acrosomal membrane prevents the conjugated lectin from adhering properly. The acrosome integrity ratio was calculated by dividing the number of spermatozoa with stained acrosomes by the total number of spermatozoa.

### 2.4. Genetic Analysis of CATSPER3 and KISS1R

Total RNA was isolated from washed sperm cell pellets obtained from semen samples using the High Pure RNA Tissue Kit (Roche Diagnostics, GmbH, Mannheim, Germany, Cat. No. 12033674001) according to the manufacturer’s protocol. To minimize RNA degradation, all procedures were performed under RNase-free conditions, and samples were immediately processed or stored at −80 °C until use. The concentration and purity of the isolated RNA were determined by measuring absorbance at 260 nm and 280 nm using a Nano-400A Micro UV Spectrophotometer (Hangzhou Allsheng Instruments Co., Ltd., Hangzhou, China). RNA quality was assessed based on the A260/A280 ratio, with values between 1.8 and 2.0 considered indicative of high purity.

Complementary DNA (cDNA) was synthesized from 1 µg of total RNA using the Transcriptor First Strand cDNA Synthesis Kit (Roche Diagnostics, GmbH, Mannheim, Germany) following the manufacturer’s instructions. The specific primer sequences for human *KISS1R* (Forward: 5′-TGCGGACCGTGACCAACTTC-3′, Reverse: 5′-CGTACCAGCGGTCCACACTC-3′), *CATSPER3* (Forward: 5′-CGAGAGCTGATGTTGGAGCA-3′, Reverse: 5′-TTGGGTCAGTGTGGCTCAAG-3′), and *ACTB* (Forward: 5′-CACCATTGGCAATGAGCGGTTC-3′, Reverse: 5′-AGGTCTTTGCGGATGTCCACGT-3′) were used to quantify gene expression levels.

Quantitative real-time PCR (qPCR) was performed using the FastStart Essential SYBR Green Master Mix (Roche Diagnostics, GmbH, Mannheim, Germany) on a LightCycler 96 system (Roche Diagnostic, GmbH, Mannheim, Germany). Specific primer pairs were designed for both target and reference genes. The amplification conditions included an initial denaturation at 95 °C for 10 min, followed by 40 cycles of denaturation at 95 °C for 15 s, annealing at 58–60 °C (depending on the primer pair) for 20 s, and extension at 72 °C for 30 s. Melting curve analysis was conducted to verify amplification specificity.

Cycle threshold (Ct) values were obtained using the LightCycler 96 quantification software (Roche Diagnostics, GmbH, Mannheim, Germany). We used the 2^ΔΔCt^ method to calculate the relative expression levels of the target gene, and these were normalized relative to the levels of the reference gene selected as β-actin.

### 2.5. Statistical Analysis

To detect a statistically significant difference in CATSPER3, KISS1R and Lectin staining (%) levels between normozoospermia and oligozoospermia groups, we conducted a prior sample size calculation using the “pwr” package in R version 4.2.1 (www.r-project.org), indicating that the minimum sample size of 26 in each arm would be required to detect a large effect size (Cohen’s d = 0.8) with 80% statistical power (1 − β) at the 5% significance level (α).

All statistical analyses were performed using R version 4.1.2. (The R Foundation for Statistical Computing, Vienna, Austria; https://www.r-project.org). To check the normality of the data and homogeneity of the variance, Shapiro–Wilk’s and Levene’s tests were run. Data were summarized as mean ± standard deviation or median with quartiles [1st quartile—3rd quartile] for staining levels and gene expression levels of CATSPER3, KISS1R and Lectin stratified to the defects such as head, tail and neck morphology, and compared these between normozoospermia and oligozoospermia groups via the Mann–Whitney U test. The effects of the relevant measurements on oligozoospermia were evaluated using multiple logistic regression analysis adjusted for age and BMI.

## 3. Results

A total of 52 patients, including 26 normozoospermic and 26 oligozoospermic patients, were included in this study with a range of ages from 18 to 50 years. The CATSPER3, KISS1R and Lectin staining levels, both overall and stratified by defect morphology of sperm according to the study groups, are given in Table 1.

**Figure 2:**
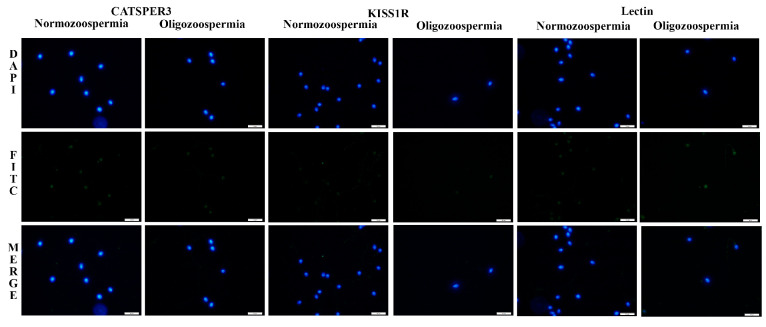
Comparison of CATSPER3, KISS1R and Lectin expression in normozoospermic and oligozoospermic individuals. 100× magnification. Blue DAPI-labeled sperm nucleus. Green FITC-labelled CATSPER3, KISS1R and Lectin expression. Merge is a combination of DAPI and FITC images. Scale bar = 10 μm.

Compared to the normozoospermic group, oligozoospermic individuals had significantly lower levels of acrosome integrity (72.42 ± 9.69 vs. 85.11 ± 10.36, *p* < 0.001, Figure 2), CATSPER3 staining (89.38 ± 7.26 vs. 95.00 ± 8.81, *p* < 0.001), and KISS1R staining (87.81 ± 7.76 vs. 96.38 ± 6.78, *p* < 0.001).

When analyzed by localization, CATSPER3 expression in the head (50.85 ± 21.28 vs. 38.92 ± 21.45, *p* = 0.031) and head–neck regions (0.62 ± 0.90 vs. 0.12 ± 0.33, *p* = 0.011) was significantly higher in the oligozoospermic group, whereas KISS1R expression in the head (51.38 ± 15.01 vs. 40.23 ± 12.46, *p* = 0.004) was also higher in oligozoospermic individuals. Conversely, KISS1R staining in the head–tail region was significantly lower in the oligozoospermic group compared to normozoospermic men (24.69 ± 15.97 vs. 46.23 ± 19.00, *p* < 0.001).

CATSPER3 staining levels were higher in the tail and head–tail regions of normozoospermic individuals compared to oligozoospermic individuals, whereas they were lower in the head region (Figure 2). Similarly, KISS1R staining levels, consistent with CATSPER3, were higher in the tail and head–tail regions of normozoospermic individuals than in oligozoospermic individuals, but lower in the head region (Figure 2).

In terms of gene expression, no statistically significant differences were observed between groups for *CATSPER3* (median [IQR]: 0.70 [0.07–7.81] vs. 1.73 [0.14–25.40], *p* = 0.519) or *KISS1R* (73.35 [50.87–142.62] vs. 68.03 [17.99–195.32], *p* = 0.405).

When the results were adjusted for age and BMI as potential confounding factors, the overall staining percentages for Lectin, CATSPER3, and KISS1R remained significantly lower in oligozoospermic patients compared to normozoospermic controls. In regional analysis of CATSPER3, the statistical difference in the head region disappeared after adjustment for age and BMI. However, the significantly lower staining levels in the tail region of oligozoospermic individuals persisted. Regarding KISS1R, the higher staining percentage in the head region of oligozoospermic men remained statistically significant after demographic adjustment. Similar to the unadjusted results, no significant changes were observed in *CATSPER3* or *KISS1R* gene expression levels after controlling for age and BMI.

## 4. Discussion

Several studies have demonstrated that members of the CATSPER channel family are expressed in both the head and flagellar regions of spermatozoa, functioning as the principal pathways for calcium (Ca^2+^) influx, which is indispensable for male fertility [20]. Consistent with previous findings, our results confirmed the robust expression of the CATSPER3 protein in human sperm cells. In terms of subcellular localization, CATSPER3 was predominantly localized in the sperm head, with additional expression observed in the neck and tail regions. This distribution pattern is in agreement with earlier reports describing CATSPER channels as integral to calcium signaling throughout the sperm structure [21,22]. Given the close functional and structural association among the CatSper family members—where the absence of even a single subunit abolishes channel function and hyperactivated motility [9]—it is essential to evaluate the specific role of CATSPER3 in altered spermatogenic conditions. A previous study investigating CATSPER2 reported that it plays a significant role in primary spermatogenic failure [23]. Furthermore, genetic analyses of *CATSPER1* polymorphisms have shown that variants such as rs2845570 are associated with an increased risk of oligozoospermia [24].

In the present study, no significant differences in gene expression levels were detected between oligozoospermic and normozoospermic individuals for *CATSPER3* or *KISS1R*. However, the expression pattern of the CATSPER3 protein in the spermatozoa of oligozoospermic men was characterized in detail and revealed critical deficiencies. While overall CATSPER3 expression was significantly higher in normozoospermic men, regional analysis in oligozoospermic spermatozoa revealed a drastic reduction, particularly in the head region. These low CATSPER3 staining levels observed in the oligozoospermic group strongly correlate with recent clinical evidence by Wang et al. (2021), who demonstrated that specific *CATSPER3* mutations directly lead to the failure of the sperm acrosome reaction [25]. The paradox of low protein staining levels despite stable *CATSPER3* gene expression suggests that this channel may be heavily regulated at the post-transcriptional or post-translational level. As highlighted by Sánchez-Jasso et al. [26] while *CATSPER3* promoter activity is regulated by specific transcription factors (CREMτ and CREBA) during spermiogenesis, the mature spermatozoon is transcriptionally silent. Thus, protein abundance relies entirely on post-translational stability [26]. Indeed, it has been previously reported that there is not always a direct relationship between protein expression and mRNA levels [27], indicating that the molecular defect in oligozoospermia might involve increased protein degradation (e.g., via the ubiquitin–proteasome system) rather than impaired gene transcription.

Acrosomal integrity was significantly reduced in oligozoospermic men compared with normozoospermic individuals (*p* < 0.001). The accurate assessment of acrosomal status is paramount in fertility evaluations. The extensive literature validates the use of specific fluorochrome-conjugated lectins (such as Peanut Agglutinin [PNA] or Pisum sativum Agglutinin [PSA]) combined with flow cytometry or quantitative fluorescence to reliably distinguish between intact and reacted acrosomes by targeting the outer acrosomal membrane glycoproteins [28,29,30,31]. Furthermore, recent advancements in in vitro induction and detection of acrosomal exocytosis underline the strong correlation between acrosomal enzyme activity and semen parameters [32,33]. To date, there are limited published studies directly linking sperm concentration with such dramatic structural acrosomal deficits. The significant loss of the CATSPER3 calcium gateway in the sperm head of our oligozoospermic cohort provides a direct mechanistic explanation for this reduced acrosomal integrity, as influx is the absolute trigger for acrosomal exocytosis.

The initiation of this calcium influx heavily relies on upstream signaling molecules, prominently the kisspeptin receptor (KISS1R). The kisspeptin system is a master regulator of the hypothalamic–pituitary–gonadal (HPG) axis [34,35], but recent evidence confirms its vital peripheral role in the urogenital system [36,37,38]. Hsu et al. demonstrated that this system is expressed not only along the HPG axis but also locally in the acrosomal region of spermatids and spermatozoa [39]. In line with these findings, our study confirmed that KISS1R expression is mainly localized in the sperm head region. Previous landmark studies by Bedford et al. [11] and by Flesch and Gadella [12] found that signaling receptors in human sperm are primarily localized in the equatorial segment, which anatomically corresponds to the head region. This is strongly supported by Pinto et al. [16] and Meccariello [40], who characterized the kisspeptin system in human spermatozoa and highlighted its role in modulating sperm functions.

However, the current literature provides limited evidence regarding the relationship between the local kisspeptin system and oligozoospermia. Zou et al. [41] reported that kisspeptin concentrations in seminal plasma are approximately 60,000 times higher than those in serum, and that higher seminal kisspeptin levels are positively correlated with sperm concentration and total sperm count. Furthermore, Hu et al. [42] emphasized the potential roles of the kisspeptin/KISS1R system in broader reproductive outcomes such as implantation. In our study, KISS1R protein expression was significantly lower in the oligozoospermic group, supporting the hypothesis that impaired kisspeptin receptor signaling may be causally associated with male subfertility and reduced acrosomal responsiveness.

The inclusion of age and BMI as covariates in our statistical model provided critical insights into the robustness of our findings. Interestingly, while the overall protein deficits remained significant, the specific localization of CATSPER3 in the head region appears to be influenced by demographic variables, as the statistical difference disappeared after adjustment. This suggests that CATSPER3 head localization might be partially modulated by systemic metabolic factors or age-related physiological changes. In contrast, the deficit of CATSPER3 in the tail region and the anomalous accumulation of KISS1R in the head region of oligozoospermic spermatozoa remained significant regardless of age or BMI. This indicates that these specific regional protein alterations are intrinsic features of the pathophysiology of oligozoospermia rather than secondary effects of aging or body weight. The persistence of higher KISS1R head staining in oligozoospermic men, despite lower overall expression, may suggest a compensatory receptor upregulation or a failure in receptor internalization/recycling in the acrosomal region [41].

Several investigations have shown that kisspeptin supplementation can promote spermatogenic cell proliferation and improve testicular function. Kisspeptin treatment has been reported to attenuate a premature acrosome reaction, early capacitation, and cellular or DNA damage [43,44]. Experimental studies by Khafaga et al. [45] demonstrated beneficial effects of kisspeptin in treating testicular degeneration in rats. Furthermore, Hsu et al. [39] observed that treatment with Kisspeptin-10 significantly increased intracellular calcium levels in spermatozoa, while other studies reported anti-apoptotic and antioxidant effects of kisspeptin on Leydig and spermatogenic cells, accompanied by elevated testosterone production [4,46]. Collectively, these findings suggest that kisspeptin is a crucial modulator of male reproductive physiology. Its high concentration in seminal fluid may enhance sperm production or function, whereas excessive or prolonged exposure could lead to receptor (KISS1R) desensitization. The simultaneous loss of KISS1R and CATSPER3 in our oligozoospermic cohort indicates a functional collapse of this critical signaling axis, reducing both cellular quality and fertilization capacity.

We note as a limitation that our protein validation relied on quantitative immunofluorescence. While this provided detailed regional localization, incorporating orthogonal assays such as Western blotting or functional Ca^2+^ imaging in future studies would further strengthen these findings. Additionally, although our sample size was statistically sufficient to detect significant differences, larger cohorts and longitudinal designs are needed to establish a direct causal relationship between the observed KISS1R-CATSPER3 downregulation and the clinical onset of oligozoospermia. Finally, while we adjusted for age and BMI, other unmeasured physiological variables could still influence sperm molecular dynamics. Addressing these points in subsequent research will be essential to fully translate these markers into clinical diagnostic tools.

## 5. Conclusions

In conclusion, this study demonstrates that the KISS1R-CATSPER3 signaling axis is significantly impaired in oligozoospermic individuals. Our results confirm that the reduction in CATSPER3 and KISS1R protein expression, along with diminished acrosomal integrity, are robust hallmarks of impaired sperm quality that persist even after adjusting for age and BMI.

The observed divergence between stable mRNA levels and decreased protein abundance suggests that these molecular deficits are primarily driven by post-translational regulation or degradation pathways. Collectively, these findings highlight the KISS1R-CATSPER3 axis as a critical determinant of acrosomal stability and fertilization potential. These proteins represent promising molecular markers for assessing male subfertility and may serve as potential targets for future diagnostic and therapeutic interventions.

## Figures and Tables

**Table 1:** The CATSPER3, KISS1R and Lectin staining (%) levels in normozoospermic and oligozoospermic patients.

	Normozoospermic (*n* = 26)	Oligozoospermic (*n* = 26)	*p*-Value	OR (95% CI)
Age (years)	31.19 ± 6.66	31.77 ± 6.28	0.749	
BMI (kg/m^2^)	26.43 ± 3.35	26.05 ± 3.77	0.702	
Lectin Staining Level (%)	85.11 ± 10.36	72.42 ± 9.69	<0.001	0.881 (0.818–0.948) *
Catsper3 Staining Level (%)	95.00 ± 8.79	89.28 ± 7.15	<0.001	0.896 (0.816–0.983) *
Head	38.90 ± 21.32	50.93 ± 21.26	0.032	1.028 (0.998–1.058)
Tail	13.21 ± 16.07	5.41 ± 6.14	0.094	0.938 (0.881–0.999) *
Neck	1.04 ± 1.34	1.34 ± 2.29	0.512	1.083 (0.798–1.470)
Kisspeptin Staining Level (%)	96.39 ± 6.82	87.86 ± 7.81	<0.001	0.817 (0.721–0.927) *
Head	40.12 ± 12.46	51.30 ± 14.92	0.004	1.069 (1.019–1.121) *
Tail	8.82 ± 12.26	3.79 ± 3.54	0.216	0.909 (0.812–1.017)
Neck	0.50 ± 0.96	1.23 ± 2.04	0.445	1.380 (0.911–2.090)
*CatSper3* gene expression (×10^3^)	1.73 [0.14–25.40]	0.70 [0.08–7.40]	0.481	0.997 (0.990–1.005)
*KISS1R* gene expression (×10^3^)	68.03 [17.99–195.32]	73.35 [50.87–142.62]	0.405	1.000 (1.000–1.000)

Data were expressed as mean ± standard deviation or median with quartiles [1st quartile–3rd quartile] and compared with the Mann–Whitney *U* test. The asterisk (*) denotes the variables that were found to be statistically significant in the multiple logistic regression analysis. Specifically, these results represent adjusted odds ratios (ORs) whose 95% confidence intervals do not include 1. All significant findings were obtained after adjustment for age and body mass index (BMI).

## Data Availability

The data presented in this study are available on reasonable request from the corresponding author. The data are not publicly available due to privacy and ethical restrictions.

## Abbreviations

The following abbreviations are used in this manuscript:

CatSper	Cation/calcium channel of spermatozoa
WHO	World Health Organization
IVF	In vitro fertilization
DNA	Deoxyribonucleic acid
KISS1	Kisspeptin
KISS1R	Kisspeptin receptor
*KISS1*	Kisspeptin gene
*KISS1R*	Kisspeptin receptor gene
IP3	İnositol triphosphate
FITC	Fluorescein isothiocyanate
PNA	Peanut glutinin
PCR	Polymerase chain reaction
HPG	Hypothalamic–pituitary–gonadal
